# Underwater endoscopic mucosal resection for circumferential adenoma of the ileocecal valve

**DOI:** 10.1055/a-2371-1066

**Published:** 2024-08-07

**Authors:** Masakatsu Fukuzawa, Sakiko Naito, Takashi Morise, Yasuyuki Kagawa, Takahiro Muramatsu, Takashi Kawai, Takao Itoi

**Affiliations:** 1 Department of Gastroenterology and Hepatology, Tokyo Medical University Hospital, Tokyo, Japan; 2 Department of Gastroenterological Endoscopy, Tokyo Medical University Hospital, Tokyo, Japan

Underwater endoscopic mucosal resection (UEMR), which was first described by Binmoeller et al. 1 , is an endoscopic procedure that reduces the risk of perforation by using buoyancy to maintain a distance from the muscularis propria. Although the en bloc resection rate for UEMR is lower than that for endoscopic submucosal dissection (ESD) for tumors of 20–30 mm in diameter, it has similar local recurrence rates, shorter procedure times, and fewer intraoperative and postoperative adverse events 2 .

An 80-year-old woman underwent a colonoscopy, which revealed a 35-mm laterally spreading tumor of the granular type on the ileocecal valve. The lesion was located on the entire circumference of the ileocecal valve, and extended approximately 2 cm into the terminal ileum ( Fig. 1 ). Based on narrow-band imaging magnification and pit pattern findings, the depth was diagnosed as cTis-T1a ( Fig. 2 ). The tumor in the ileum was diagnosed as an adenoma, and thus piecemeal resection by UEMR was performed ( Video 1 ).

**Fig. 1:**
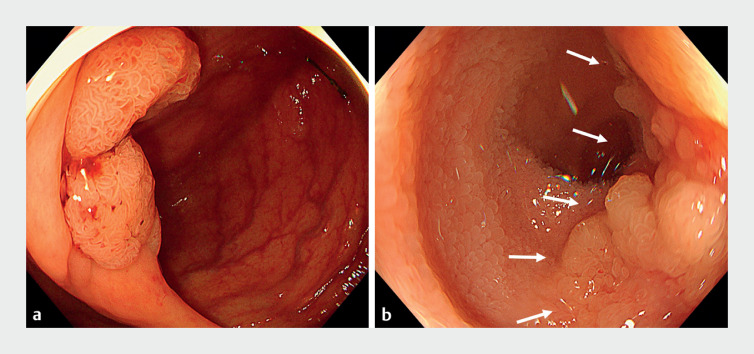
Endoscopic images of the tumor. **a** A 35-mm laterally spreading tumor on the circumference of the ileocecal valve. **b** The tumor was observed over the entire circumference of the ileocecal valve, and extended approximately 2 cm into the ileum (white arrows).

**Fig. 2:**
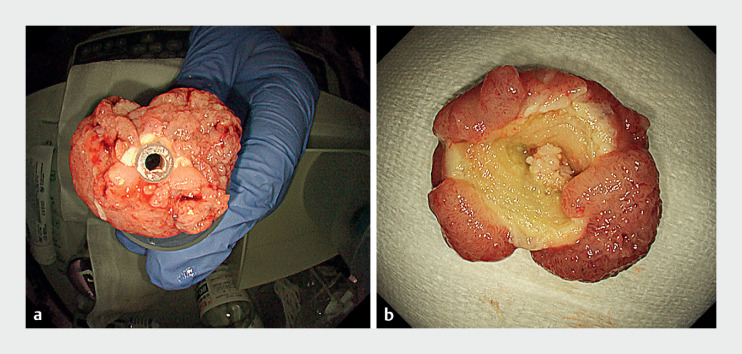
Specimen resected by underwater endoscopic mucosal resection. **a** Luminal side of the resected specimen. **b** Wall side of the resected specimen.

Underwater endoscopic mucosal resection for circumferential adenoma of the ileocecal valve.Video 1

A 33-mm snare was used to resect the nodule on the ileocecal valve en bloc, whereas piecemeal resection was performed on the residual tumors at the resection margins and in the ileum ( Fig. 3 ). The procedure took 25 minutes, with no intraoperative or postoperative complications being observed.

**Fig. 3:**
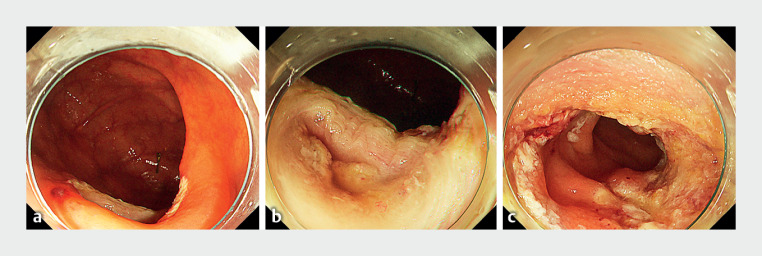
Endoscopic images after underwater endoscopic mucosal resection with polypectomy. **a** The ileocecal valve. **b** Post-treatment ulcer in the ileocecal valve (close-up view). **c** Post-treatment ulcer in the terminal ileum.

Pathological analysis confirmed adenocarcinoma with adenoma (tub1–tub2), pTis (M), Ly0, V0, pHMX, and VM0. Surveillance endoscopy at 3 months post-treatment detected a small residual tumor, prompting a hot biopsy. Owing to stenosis in the ileocecal valve, balloon dilation was required to insert the scope. No residual lesion was found on the scar post-treatment, and no tumor components were identified on pathological analysis of the biopsy.

En bloc resection using ESD is difficult for lesions extending from the ileocecal valve into the ileum, making UEMR a viable and safer alternative with shorter procedure times.

Endoscopy_UCTN_Code_TTT_1AQ_2AD_3AC

## Notes

Endoscopy E-Videos https://eref.thieme.de/e-videos
*E-Videos* is an open access online section of the journal *Endoscopy* , reporting on interesting cases and new techniques in gastroenterological endoscopy. All papers include a high-quality video and are published with a Creative Commons CC-BY license. Endoscopy E-Videos qualify for HINARI discounts and waivers and eligibility is automatically checked during the submission process. We grant 100% waivers to articles whose corresponding authors are based in Group A countries and 50% waivers to those who are based in Group B countries as classified by Research4Life (see: https://www.research4life.org/access/eligibility/ ). This section has its own submission website at https://mc.manuscriptcentral.com/e-videos .
